# Supplementary material for: Trim33 masks a non-transcriptional function of E2f4 in replication fork progression
Source: Nat Commun. 2023 Aug 23;14:5143. doi: 10.1038/s41467-023-40847-0 (PMC10447549; doi:10.1038/s41467-023-40847-0)
Supplement: Supplementary file 1 — Supplementary Information [file 41467_2023_40847_MOESM1_ESM.pdf]

Trim33 masks a non-transcriptional function of E2f4 in replication fork progression

Vanessa Rousseau, Elias Einig, Chao Jin, Julia Horn, Mathias Riebold, Tanja Poth, Mohamed-ali Jarboui, Michael Flentje, Nikita Popov*

* Correspondence: nikita.popov@med.uni-tuebingen.de

Supplementary Information

Supplementary Figures

Supplementary Figure 1

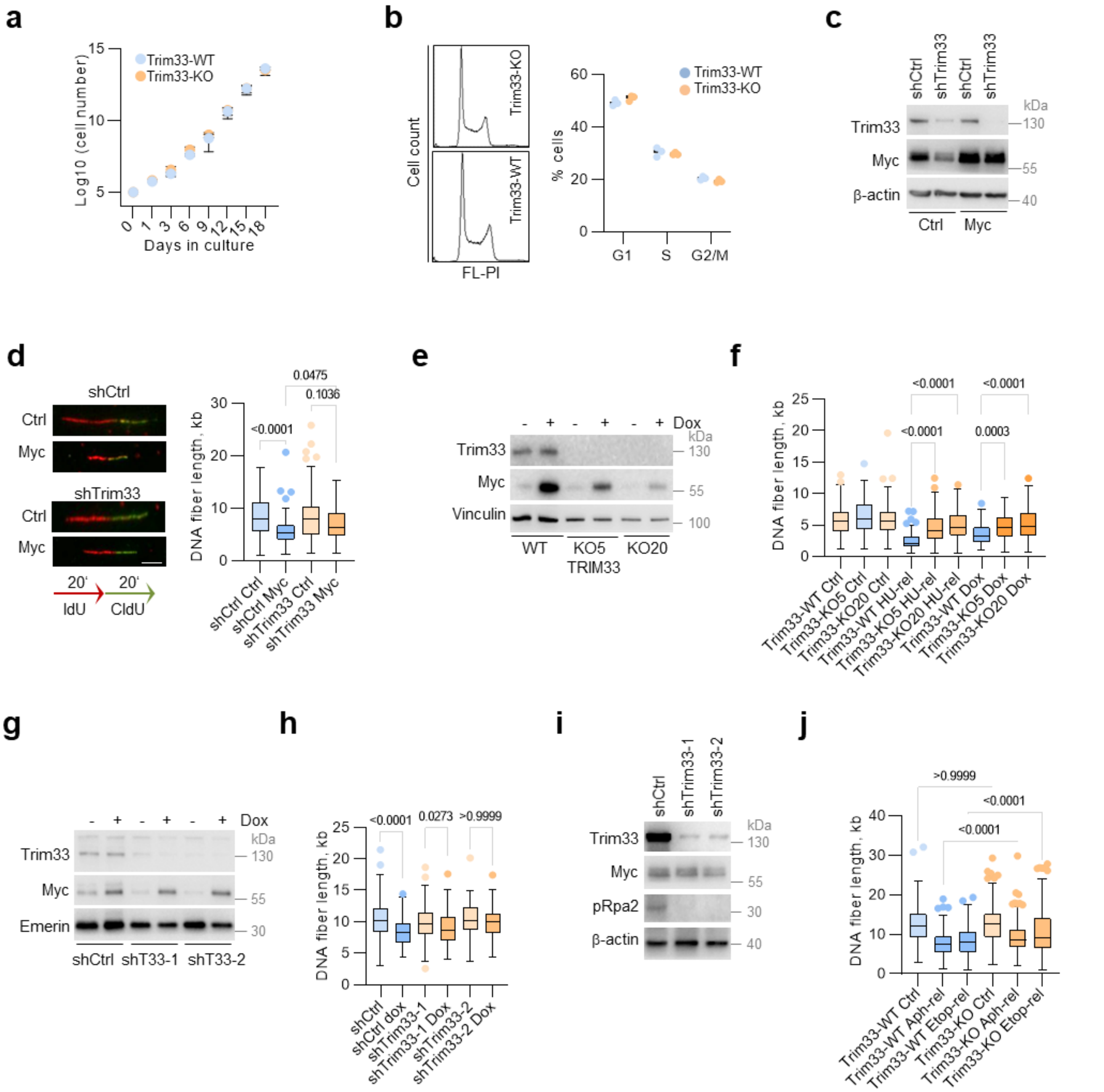

Supplementary Figure 1. Loss of Trim33 abates replicative stress. a) Growth curve for p19Nras Trim33-WT and Trim33-KO cells under standard culture conditions; n=2. **b)** PI-FACS profiles and cell cycle distribution of Trim33-WT and Trim33-KO cells cultured under standard conditions; n=3. **c)** Immunoblotting analysis of p19/Nras and p19/Nras/Myc cells expressing shCtrl or shTrim33; n=3. **d)** DNA fiber assays in p19/Nras and p19/Nras/Myc cells expressing shCtrl or shTrim33; n = 3. 100 fibers were measured and significance was determined using Kruskal-Wallis test followed by Dunn's multiple comparison. Scale bar = 1 µm. **e)** Immunoblotting analysis of Trim33, Myc and vinculin in U2OS Trim33-WT and two Trim33-KO cell lines, expressing a doxycycline-inducible Myc allele with or without doxycycline (dox) treatment. **f)** DNA

fiber assays in U2OS cells shown in E) with or without doxycycline treatment or during release from a 4h HU treatment; n=3. 100 fibers were measured. Significance was determined using Kruskal-Wallis test followed by Dunn‘s multiple comparison. **h)** Immunoblotting analysis of U2OS cells, expressing two Trim33 shRNAs with or without doxycycline (dox) treatment; n=2. **h)** DNA fiber assays in U2OS cells, expressing shCtrl or shTrim33, without or with doxycycline treatment, labelled with IdU/CldU for 40 min; n = 2. 100 fibers were measured. Significance was determined using Kruskal-Wallis test followed by Dunn‘s multiple comparison. **i)** Immunoblotting analysis in p19/Nras/Myc cells expressing shRNAs against Trim33 or a control shRNA; n=3. **j)** DNA fiber assays in Trim33-WT and Trim33-KO cells p19Nras cells during release from aphidicolin (Aph-rel) or etoposide (Etop-rel) treatment; n=3. 100 fibers were measured; significance was determined by Kruskal-Wallis test with Dunn‘s multiple comparison. **d,f,h,j**) Boxplots represent median±quartiles with whiskers ranging up to 1.5-fold of the interquartile range. Source data are provided as a Source Data file.

Supplementary Figure 2

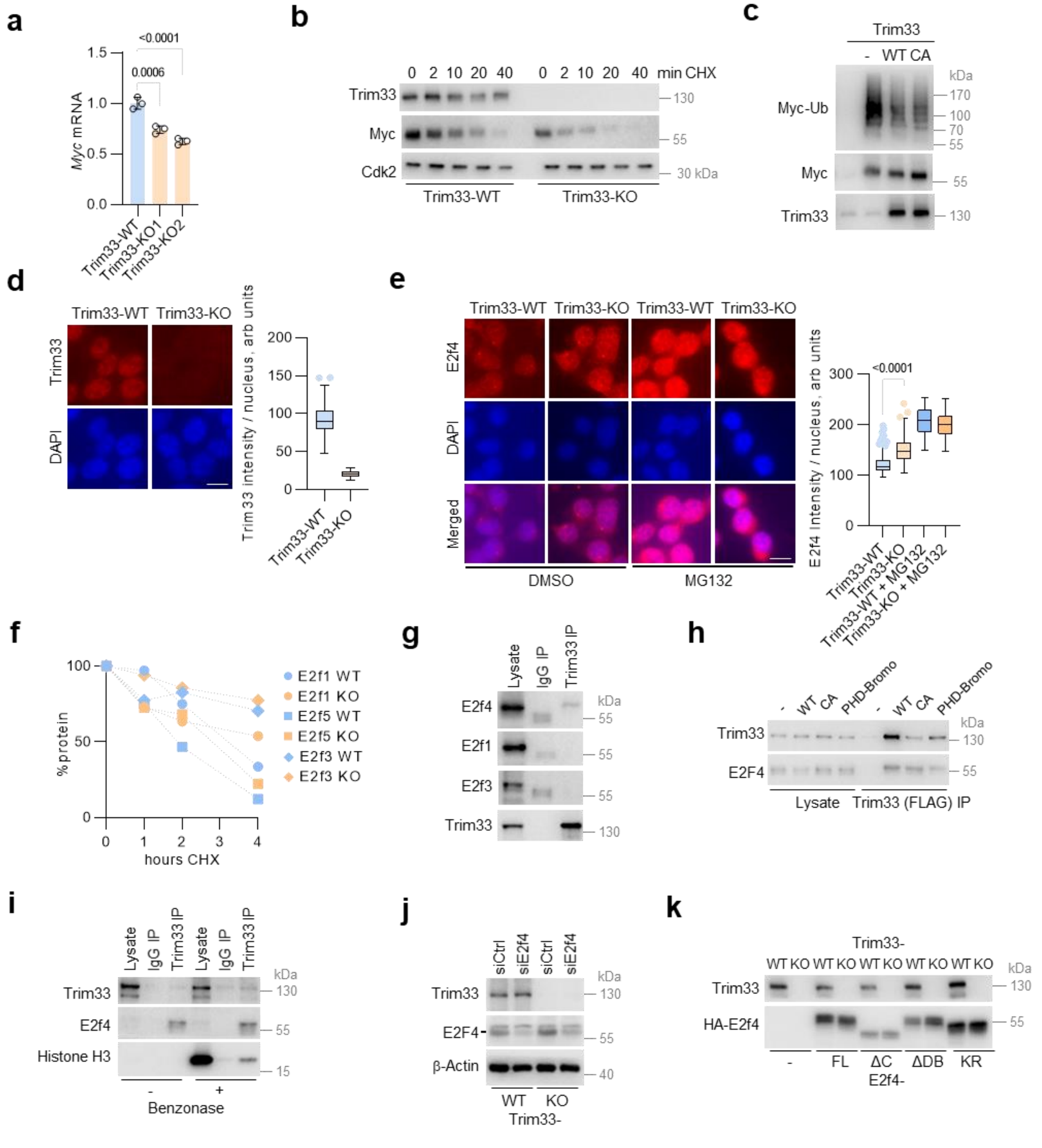

Supplementary Figure 2. Deletion of Trim33 stabilizes E2f4. a) qPCR analysis of Myc expression in p19/Nras Trim33-WT and Trim33-KO cells (mean of three technical replicates±SD); Significance was determined using one-way Anova followed by Dunnett's multiple comparison with the control sample. A representative of three independent experiments is shown (n=3). **b)**

Cycloheximide chase assays in p19/Nras Trim33-WT and Trim33-KO cells; n=3. **c)** Ubiquitin pulldown assays in HeLa cells transfected with vectors encoding Myc, his-Ub and wildtype (WT) or catalytically inactive (CA) Trim33; n=2. **d)** Immunofluorescence analysis of p19/Nras Trim33-WT and Trim33-KO; n=3. Scale bar = 5 µm. **e)** Immunofluorescence analysis of p19/Nras Trim33-WT and Trim33-KO cells treated with 10 µM MG132 or DMSO control for 8 h; n=3. Scale bar = 5 µm. Significance was determined using Kruskal-Wallis test followed by Dunn's multiple comparison. **f)** Quantification of cycloheximide chase assays in Trim33-WT and Trim33-KO p19/Nras with the indicated antibodies; n=2. **g)** Immunoprecipitation assays with Trim33 antibodies in U2OS cells; n=1. **h)** Immunoprecipitation assays in HeLa cells transfected with vectors for HA-tagged E2f4 and the indicated Flag-tagged Trim33 variants; n=2. i**)** Immunoprecipitation analysis with E2f4 antibodies ± benzonase treatment of lysates of p19/Nras Trim33-WT cells; n=3. **j)** Immunoblotting analysis in p19/Nras Trim33-KO and Trim33-WT cells transfected with siRNA pool against E2f4 or control (siCtrl); n=3. **k)** Immunoblotting analysis of p19/Nras Trim33-WT and Trim33-KO cells expressing the indicated E2f4 variants; n=2. **d,e**) Boxplots represent median±quartiles with whiskers ranging up to 1.5-fold of the interquartile range. Source data are provided as a Source Data file.

Supplementary Figure 3

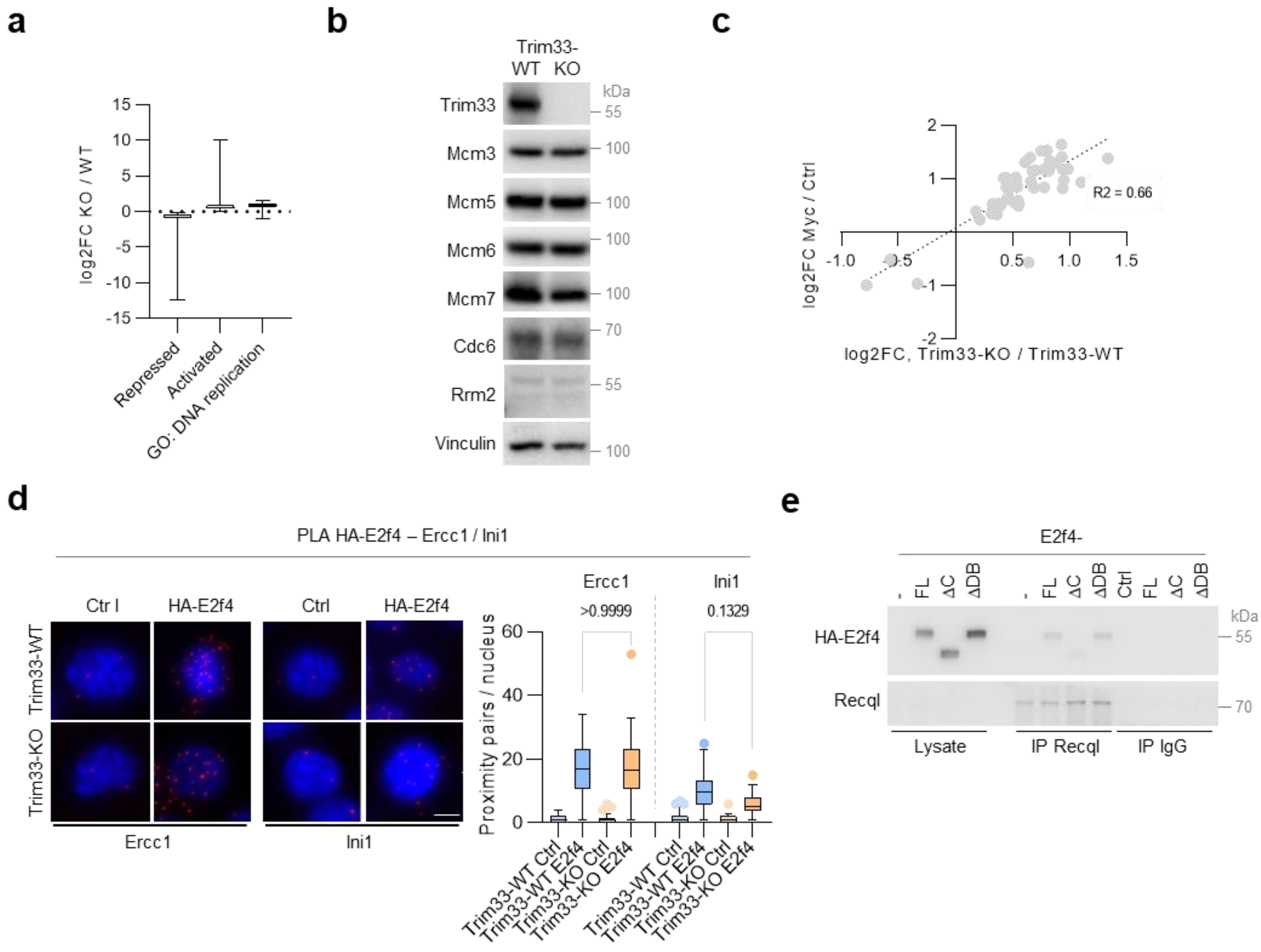

Supplementary Figure 3. E2f4 target genes and interaction partners. a) Median regulation of up- and downregulated genes in p19/Nras Trim33-KO cells relative to Trim33-WT cells. Boxplots represent median values ±quartiles and whiskers range from maximum to minimum values for average regulation in two independent RNA-seq experiments. **b)** Immunoblotting analysis of lysates of p19/Nras Trim33-WT and Trim33-KO cells with the indicated antibodies; n=3. **c)** Regulation of replication-associated genes (GO:0006260) in p19/Nras Trim33-KO cells relative to Trim33-WT cells plotted versus regulation in p19/Nras Trim33-WT Myc-expressing cells relative to Ctrl vector-expressing cells. **d)** PLA analysis of E2f4 interaction with Ercc1 and Ini1 in p19/Nras Trim33-WT and Trim33-KO cells stably expressing HA-tagged E2f4 or a Ctrl vector; n=2. Scale bar=2 µm. Significance was determined using Kruskal-Wallis test followed by Dunn‘s multiple comparison. 50 cells were analyzed per condition. Boxplots represent median±quartiles with whiskers ranging up to 1.5-fold of the interquartile range. **e)** Immunoprecipitation of endogenous

Recql from formaldehyde-crosslinked p19/Nras Trim33-KO cells, stably expressing the indicated HA-E2f4 proteins; n=2. Source data are provided as a Source Data file.

Supplementary Figure 4

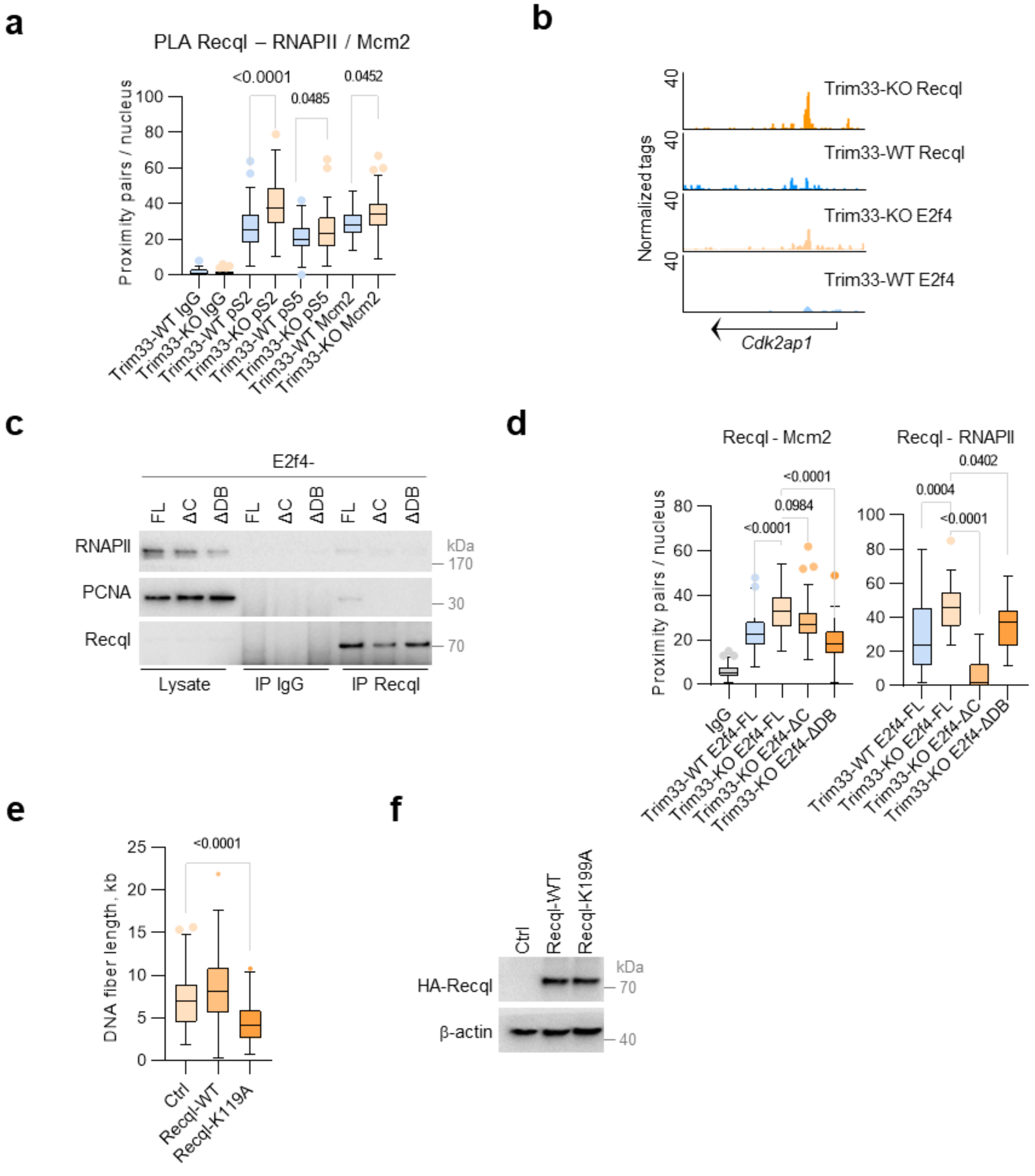

Supplementary Figure 4. E2f4 promotes Recql binding to chromatin. a) PLA assays in p19/Nras Trim33-WT and Trim33-KO cells with antibodies to Recql and pS5- or pS2-RNAPII, Mcm2 or a control IgG. From left, n = 44,50,50,50,50,50,28,50 cells were analyzed using two-tailed, unpaired t-test independently for each antibody pair. **b)** Representative genome browser tracks of Cut&Run analysis of HA-Recql binding in Trim33-WT and Trim33-KO cells expressing HA-tagged Recql at an intergenic E2f4 binding site. Lower two tracks show E2f4 ChIP-seq. **c)** Immunoprecipitation analysis with Recql antibodies in p19/Nras Trim33-KO cells expressing E2f4-FL, E2f4-ΔC or E2f4-ΔDB; n=2. **d)** PLA assays with antibodies to Recql and Mcm2 (from

left, n = 109,148,70,100,141 cells) or RNAPII (from left, n = 47,42,50,45 cells) in Trim33-KO cells expressing the indicated E2f4 variants. Kruskal-Wallis test followed by Dunn‘s multiple comparison. **e)** DNA fiber assays in p19/Nras Trim33-KO cells expressing Recql-WT and Recql-K119A after release from HU treatment; n=2. Significance was determined using Kruskal-Wallis test followed by Dunn‘s multiple comparison. n = 100 fibers per group. **f)** Immunoblot documenting expression of Recql variants in p19/Nras Trim33-KO cells, used in e). **a,d,e**) Boxplots represent median±quartiles with whiskers ranging up to 1.5-fold of the interquartile range. Source data are provided as a Source Data file.

Supplementary Figure 5

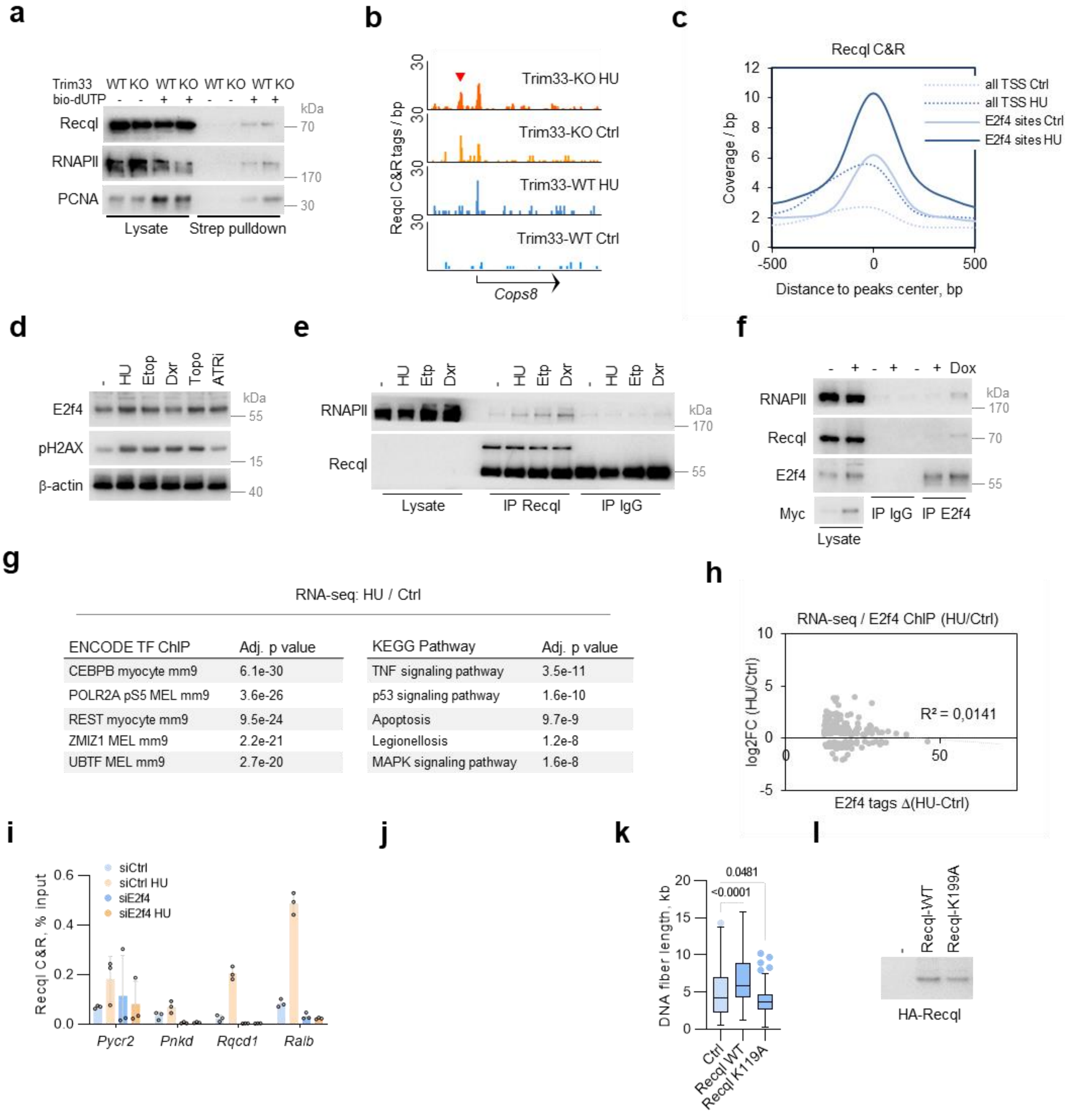

ENCODE TF ChIP	Adj. p value
CEBPB myocyte mm9	6.1e-30
POLR2A pS5 MEL mm9	3.6e-26
REST myocyte mm9	9.5e-24
ZMIZ1 MEL mm9	2.2e-21
UBTF MEL mm9	2.7e-20

KEGG Pathway	Adj. p value
TNF signaling pathway	3.5e-11
p53 signaling pathway	1.6e-10
Apoptosis	9.7e-9
Legionellosis	1.2e-8
MAPK signaling pathway	1.6e-8

Supplementary Figure 5. Replicative stress triggers Recql recruitment to chromatin. a) Nascent chromatin capture assay in p19/Nras Trim33-WT and Trim33-KO cells following release from a 4hr HU treatment; n=2. **b)** Representative genome browser tracks for HA-Recql Cut&Run in p19/Nras Trim33-WT and Trim33-KO cells before and after exposure to HU showing selective recruitment of Recql to chromatin in Trim33-WT cells. The pointer indicates a peak, specific to Trim33-KO cells. **c)** Sequencing tag coverage for HA-Recql Cut&Run signal in p19/Nras Trim33-WT cells before and after exposure to HU at all TSS and at E2f4 sites. **d)** Immunoblotting analysis

of E2f4 levels following a 4hr treatment with different genotoxins in p19/Nras Trim33-WT cells. Etop – etoposide (5uM), Dxr – doxorubicin (5uM), ATRi - ATR inhibitor AZD6738 (10uM), Topo – topotecan (0.1uM); n=2. **e)** Immunoprecipitation of endogenous Recql in p19Nras Trim33-Wt cells treated with HU, Etoposide or doxorubicin; n=2. **f)** Immunoprecipitation of endogenous E2f4 in U2OS cells before and after induction of Myc with doxycycline; n=2. **g)** Gene set enrichment analysis of RNA-seq data from untreated and HU-treated (4hr) p19/Nras Trim33-WT cells (1170 genes at p adj < 0.05, any log2FC). The data were analysed using the Enrichr portal against the ENCODE TF and KEGG pathway datasets; top 5 enriched datasets are shown. **h)** Linear regression analysis of E2f4 binding (normalized tags) and mRNA expression changes (log2FC) in HU-treated versus untreated p19/Nras Trim33-WT cells for 189 genes, which showed E2f4 recruitment after HU. **i)** Cut&Run-qPCR analysis of endogenous Recql in p19/Nras Trim33-WT and Trim33-KO cells transfected with siRNA against E2f4 or a control siRNA at the indicated TSS regions. Mean values of percent input and standard deviations are plotted for three technical replicates. **J)** DNA fiber assays in p19/Nras Trim33-WT, expressing indicated E2f4 variants, after release from HU treatment. 140 fibers were counted per condition; n = 2.These data are also shown in Figure 2J. **k)** DNA fiber assays in Trim33-WT cells expressing Recql-WT and Recql-K119A proteins, following release from a 4hr HU treatment. 100 fibers were counted per sample and the data were analyzed using Kruskal-Wallis test and Dunn‘s multiple comparison. **l)** Immunoblots documenting expression of Recql variants in p19/Nras Trim33-WT cells; n=3**. j,k)** Boxplots represent median±quartiles with whiskers ranging up to 1.5-fold of the interquartile range. Source data are provided as a Source Data file.

Supplementary Figure 6

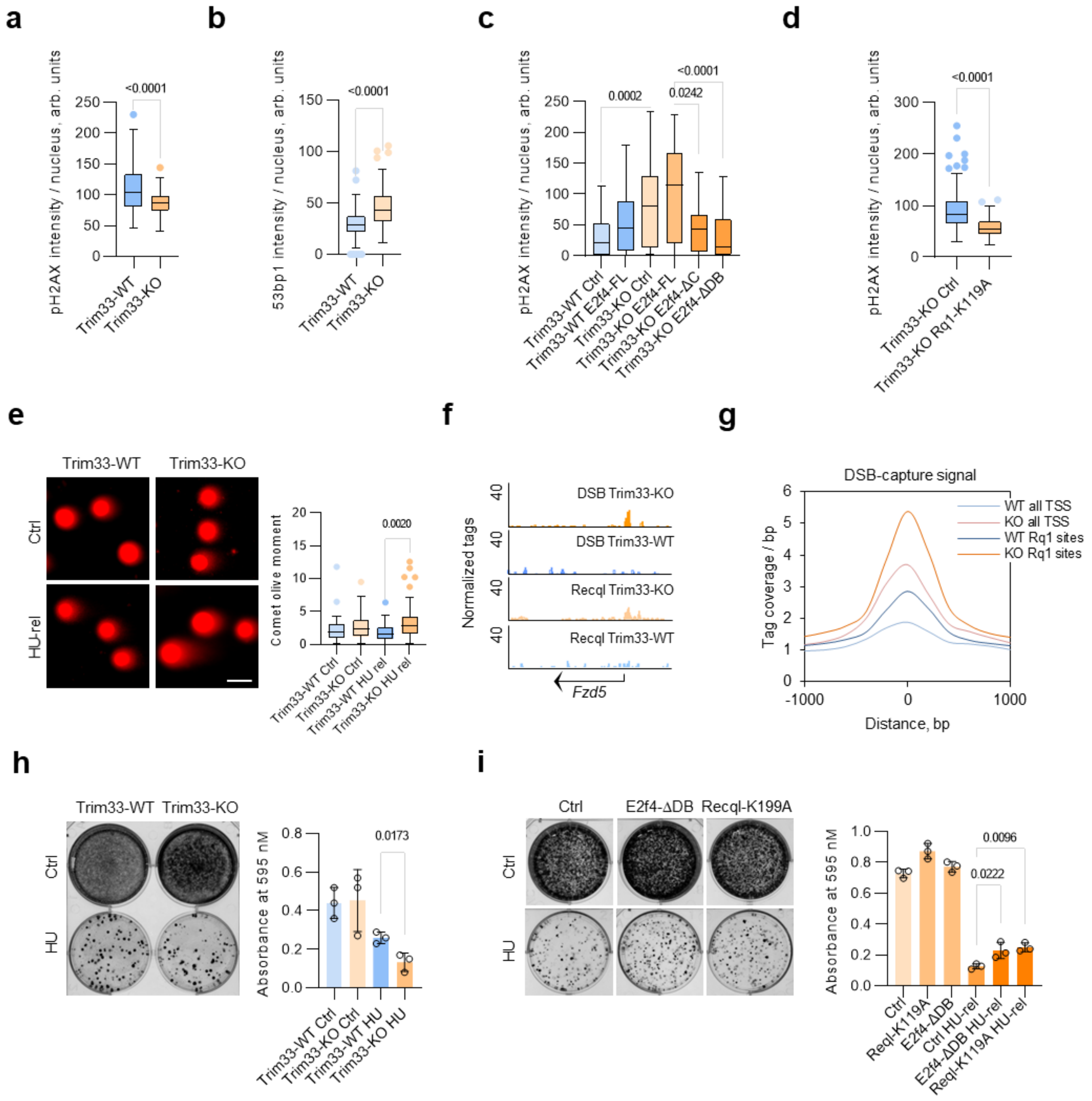

Supplementary Figure 6. Deletion of Trim33 increases DNA damage during recovery from replicative stress. a) Immunofluorescence staining with pH2AX antibody in unchallenged p19/Nras Trim33-WT and Trim33-KO cells, n=3 experiments. From left, n = 123,118 cells. Significance was determined with the non-parametric, two-tailed Mann-Whitney test. **b)** Quantification of total 53bp1 immunofluorescence signal in p19/Nras Trim33-WT and Trim33-KO cells cultured in the presence of 0.1 mM HU. From left, n = 231,228 cells. significance was determined with the non-parametric, two-tailed Mann-Whitney test. **c)** Immunofluorescence staining with pH2AX antibody in p19/Nras Trim33-WT and Trim33-KO cells expressing the

indicated E2f4 variants after 24h release from HU. From left, n = 51,54,51,53,54,49 cells. The data were analyzed by Kruskal-Wallis test and Dunn's multiple comparison. **d)** Immunofluorescence staining with pH2AX antibody in p19/Nras Trim33-KO cells expressing the Recql-K119A or a control vector at 24h after release from HU treatment; n=2. The data were analyzed with the non-parametric, two-tailed Mann-Whitney test. From left, n = 324,94 cells. **e)** Neutral comet assays in p19/Nras Trim33-WT and Trim33-KO cells transfected with the indicated siRNAs, untreated or release from a 24h HU treatment. Scale bar = 20 µM. From left, n = 26,57,38,95 cells. Significance was determined as in c). **f)** Genome browser tracks for HA-Recql Cut&Run (+HU) and DSB capture at 24 hours post-HU in p19/Nras Trim33-WT and Trim33-KO cells. g**)** Sequencing tag coverage for DSB capture signal in Trim33-WT and Trim33-KO cells at all TSS and at Recql sites (Rq1) at 24 hours post HU treatment. **h)** Colony formation assays in untreated and HU-treated p19Nras Trim33-WT and Trim33-KO cells. Quantification of crystal violet staining of three biological replicates (mean±SD). Significance was determined by an unpaired, two-tailed t test. **i)** Colony formation assays in untreated and HU-treated p19Nras Trim33-KO cells, expressing E2f4-dDB or Recql-K119A proteins. Quantification of crystal violet staining for three biological replicates (mean±SD). Significance was determined using one-way Anova with Dunnett's multiple comparison with the Ctrl HU-rel sample. **a,b,c,d,e)** Boxplots represent median±quartiles with whiskers ranging up to 1.5-fold of the interquartile range. Source data are provided as a Source Data file.

Supplementary Figure 7

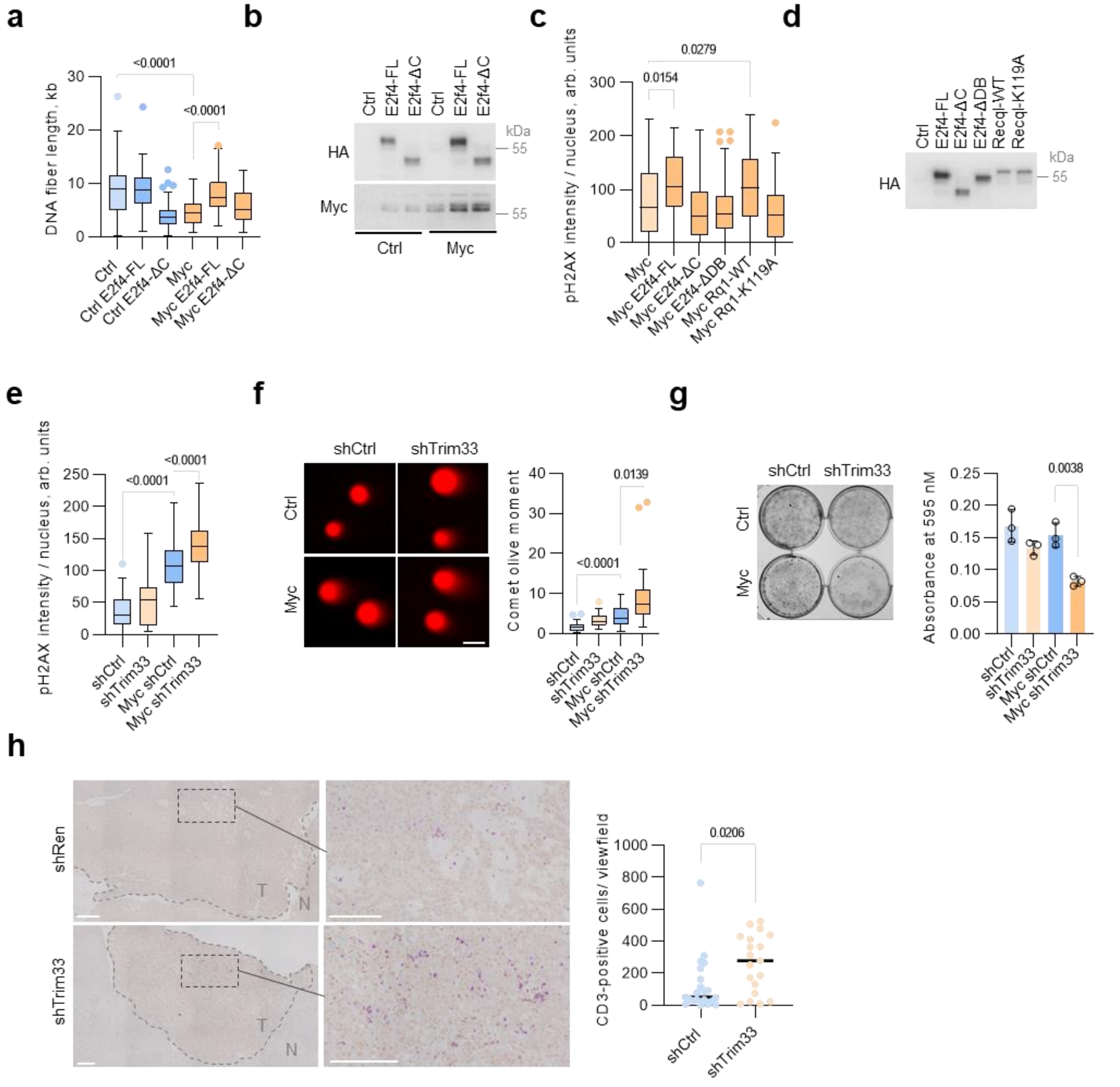

Supplementary Figure 7. Deletion of Trim33 promotes Myc-induced DNA damage and delays liver tumorigenesis. a) DNA fiber assays in p19KO/Nras and p19KO/Nras/Myc cells expressing the indicated E2f4 variants; n = 3. 100 fibers were measured; significance was determined using Kruskal-Wallis test and Dunn‘s multiple comparison. **b)** Immunoblotting analysis of p19/Nras and p19KO/Nras/Myc cells expressing the indicated E2f4 variants, shown in a). **c)** Immunofluorescence analysis with pH2AX antibodies in p19/Nras and p19/Nras/Myc cells expressing the indicated E2f4 and Recql variants. From left, n = 97,45,48,92,57,45 cells. Significance was determined as in a). **d)** Immunoblotting analysis showing expression of E2f4 and Recql variants in cells shown in c). **e)** Immunofluorescence analysis with pH2AX antibodies in p19/Nras/Myc cells expressing shCtrlor shTrim33. From left, n = 86,24,179,132 cells. The data

were analyzed as in a). **f)** Neutral comet assays in Trim33-WT and Trim33-KO cells untreated or released for 24h from HU treatment. Scale bar = 20 µm. From left, n = 39,34,25,27 cells. The data were analyzed as in a). **g)** Colony formation assay in p19/Nras and p19/Nras/Myc cells, expressing shCtrl or shTrim33. Quantification of crystal violet staining of three biological replicates (mean±SD). Significance was determined by Anova with Tukey's multiple comparison. **h)** Representative images of immunohistochemistry analysis with CD3 antibodies on FFPE sections of tumor-bearing livers;. Scale bar = 200 µm. Quantification of CD3-positive cells per field of view in tumors expressing shTrim33 or shCtrl. Significance was determined by an unpaired, two-tailed t-test. **a,c,e,f)** Boxplots represent median±quartiles with whiskers ranging up to 1.5-fold of the interquartile range. Source data are provided as a Source Data file.

Supplementary Tables

Supplementary Table 1. Antibodies used in the study

**Antigen**	**Vendor**	**Catalogue number**
Biotin	Santa Cruz	sc-101339
BrdU (BU1/75 / ICR1)	Abcam	ab6326l
BrdU (Bu20a)	Cell Signaling	5292S
53bp1	Novus	NB100-304SS
beta Actin (AC-15)	Santa Cruz	sc-69879
Cdc6 (C42F7)	Cell Signaling	3387
Cdk2 (D-12)	Cell Signaling	sc-6248
pS824-Kap1	Bethyl	A300-767A
pS317-Chk1	Cell Signaling	12302
pS4/8-Rpa2	Bethyl	a300-245A
E2f4	Proteintech	10923-1-AP
Ercc1 (D-10)	Santa Cruz	sc-17809
Ini1 (A-5)	Santa Cruz	sc-166165
Emerin (H-12)	Santa Cruz	sc-25284
FLAG (M2)	Sigma	F3165
Histone H3 (96C10)	Cell Signaling	3638P
Histone H4 (L64C1)	Cell Signaling	2935P
HA-Tag (6E2)	Cell Signaling	2367S
HA-Tag (C29F4)	Cell Signaling	3724S
mouse (G3A1) mAb IgG1 Isotype Control	Cell Signaling	5415
rabbit (DA1E) mAb IgG XP Isotype Control	Cell Signaling	3900

mouse IgG, Alexa Fluor 488 Conjugate	Cell Signaling	4408
mouse IgG, Alexa Fluor 555 Conjugate	Cell Signaling	4409
rabbit IgG, Alexa Fluor 488 Conjugate	Cell Signaling	4412
rabbit IgG, Alexa Fluor 555 Conjugate	Cell Signaling	4413
rat IgG, Alexa Fluor 488 Conjugate	Cell Signaling	4416
Mcm2 (D7G11)	Cell Signaling	3619
Mcm3 (E-8)	Santa Cruz	sc-390480
Mcm5	Abcam	ab17967
Mcm6 (H-8)	Santa Cruz	sc-393618
Mcm7	Bethyl	A302-585A
mouse IgG, HRP conjugate	Cell Signaling	7076
rabbit IgG, HRP conjugate	Cell Signaling	7074
c-Myc (C-33)	Santa Cruz	sc-42
c-Myc (D3N8F)	Cell Signaling	13987
c-Myc (Y69)	Abcam	ab32072
pS139-H2AX (20E3)	Cell Signaling	9718
pH2AX (ser139)	Santa Cruz	sc-517348
PCNA (PC-10)	Santa Cruz	sc-56
Recql	Bethyl	A300-447
Recql	Invitrogen	PA5-27100
Recql	Abcam	ab151501
Recql (A-9)	Santa Cruz	sc-166388

Rrm2 (A-5)	Santa Cruz	sc-398294
RNAPII (D8L4Y)	Cell Signaling	14958
pS2-RNAPII	Abcam	ab5095
pS5-RNAPII (D9N5I)	Cell Signaling	13523
Trim33	Sigma	HPA004345
Trim33 (6D1)	Sigma	WH0051592M1
Vinculin (V824)	Sigma	SAB4200080
GST Tag	Bethyl	A190-122A
E2f4 (GG22-2A6)	Merck	05-312

Supplementary Table 2. Oligonucleotides used in the study

Name	Sequence
shmTrim33_1 for	CCGGTCGATACCAAACTACTATAAACTCGAGTTTATAGTAGTTTGGTATCGATTTTTG
shmTrim33_1 rev	AATTCAAAAATCGATACCAAACTACTATAAACTCGAGTTTATAGTAGTTTGGTATCGA
shmTrim33_2 for	CCGGCGTGTGATAGATTGACGTGTACTCGAGTACACGTCAATCTATCACACGTTTTTG
shmTrim33_2 rev	AATTCAAAAACGTGTGATAGATTGACGTGTACTCGAGTACACGTCAATCTATCACACG
TRIM33 sh1 f	CCGGCAAACGAGTAGAACAGGAAACTCGAGTTTCCTGTTCTACTCGTTTGTTTTTG
TRIM33 sh1 r	AATTCAAAAACAAACGAGTAGAACAGGAAACTCGAGTTTCCTGTTCTACTCGTTTG
TRIM33 sh2 f	CCGGACCCTTATCAATGAAATTAACTCGAGTTAATTTCATTGATAAGGGTTTTTTG
TRIM33 sh2 r	AATTCAAAAAACCCTTATCAATGAAATTAACTCGAGTTAATTTCATTGATAAGGGT
E2f4-FL for	TGAGTCGGCCGGTGGATCCAATGTACCCTTACGACGTG
E2f4-FL rev	GGCGGATCCGTCGACACTAGTCAGAGGTTGAGAACAGG
E2f4-ΔC for	TGAGTCGGCCGGTGGATCCAATGTACCCTTACGACGTG
E2f4-ΔC rev	GGCGGATCCGTCGACACTAGTCACTCAAAGGAGGTAGAAGGGTTG
E2f4-ΔDB for	CTAGCTGTACGCCAGAAGGCAGCAATAGCAGCAATTACCAATGTTTTGGAAG
E2f4-ΔDB rev	CTTCCAAAACATTGGTAATTGCTGCTATTGCTGCCTTCTGGCGTACAGCTAG
E2f4-KR for	TGAGTCGGCCGGTGGATCCAATGTACCCATACGATGTTCCAGATTACGCTGCAGAGGCAGGACCGCAAGCT
E2f4-KR Rev	GGCGGATCCGTCGACACTAGTCAAAGGTTGAGGACGGG
Recql-WT for	TGAGTCGGCCGGTGGATCCAATGTACCCTTACGACGTGCCCGACTACGCCGGGGCGTCCGTTTCAGCTCTAAC

Recql-WT rev	GAGGGGCGGATCCGTCGACATCAGGCATCATCGATTTTTCTTTTC
Recql-K119A for	CTACAGGAGGTGGAGCGAGCTTATGTTACCAG
Recql-K119A rev	CTGGTAACATAAGCTCGCTCCACCTCCTGTAG
sgTrim33_1 for	CACCGTAGCGCGCCGGTAACCGCCG
sgTrim33_1 rev	AAACCGGCGGTTACCGGCGCGCTAC
sgTrim33_2 for	CACCGTCCACCAGCACCGCGGCGAG
sgTrim33_2 rev	AAACCTCGCCGCGGTGCTGGTGGAC
sgTrim33_3 f	CACCGTCTCGCCGCGGTGCTGGTGG
sgTrim33_3 r	AAACCCACCAGCACCGCGGCGAGAC
TRIM33 ED887AA f	GATGATGACCCAAATGCCGCCTGGTGTGCTGTCTGC
TRIM33 ED887AA r	GCAGACAGCACACCAGGCGGCATTTGGGTCATCATC
TRIM33 FN1038AA f	GAACTGTGAAAGGGCAGCCGAAATGATGAAAG
TRIM33 FN1038AA r	CTTTCATCATTTCGGCTGCCCTTTCACAGTTC
E2f4 pGEX4t3 f	AtcggatctggttccgcgtgGGGCGGAGGCCGGGCCACAG
E2f4 pGEX4t3 FL f	tcacgatgcggccgctcgagTCAGAGGTTGAGAACAGGCACATCAAAGAGGTCAC
E2f4 pGEX4t3 1-105 r	TcacgatgcggccgctcgagTCACTCGATCTCTGCCTTGAGCTCAATCAGTTTGTCAGC
E2f4 pGEX4t3 300-C f	AtcggatctggttccgcgtgGGCAGTCTTCTGCCCTGCTG
E2f4 90-200 f	AtcggatctggttccgcgtgGGACCCGGGAGATTGCTGAC
E2f4 90-200 r	TcacgatgcggccgctcgagTCACCATGCCTCCTTGTTCAC
