# Supplementary material for: Trim33 masks a non-transcriptional function of E2f4 in replication fork progression
Source: Nat Commun. 2023 Aug 23;14:5143. doi: 10.1038/s41467-023-40847-0 (PMC10447549; doi:10.1038/s41467-023-40847-0)
Supplement: Supplementary file 3 — Reporting Summary [file 41467_2023_40847_MOESM3_ESM.pdf]

nature portfolio

Corresponding author(s): Nikita Popov

Last updated by author(s): Aug 1, 2023

Reporting Summary

Nature Portfolio wishes to improve the reproducibility of the work that we publish. This form provides structure for consistency and transparency in reporting. For further information on Nature Portfolio policies, see our Editorial Policies and the Editorial Policy Checklist.

Statistics

For all statistical analyses, confirm that the following items are present in the figure legend, table legend, main text, or Methods section.

n/a	Confirmed	
☐	☒	The exact sample size (*n*) for each experimental group/condition, given as a discrete number and unit of measurement
☒	☐	A statement on whether measurements were taken from distinct samples or whether the same sample was measured repeatedly
☐	☒	The statistical test(s) used AND whether they are one- or two-sided<br>*Only common tests should be described solely by name; describe more complex techniques in the Methods section.*
☒	☐	A description of all covariates tested
☒	☐	A description of any assumptions or corrections, such as tests of normality and adjustment for multiple comparisons
☐	☒	A full description of the statistical parameters including central tendency (e.g. means) or other basic estimates (e.g. regression coefficient) AND variation (e.g. standard deviation) or associated estimates of uncertainty (e.g. confidence intervals)
☐	☒	For null hypothesis testing, the test statistic (e.g. *F*, *t*, *r*) with confidence intervals, effect sizes, degrees of freedom and *P* value noted<br>*Give P values as exact values whenever suitable.*
☒	☐	For Bayesian analysis, information on the choice of priors and Markov chain Monte Carlo settings
☒	☐	For hierarchical and complex designs, identification of the appropriate level for tests and full reporting of outcomes
☐	☒	Estimates of effect sizes (e.g. Cohen's *d*, Pearson's *r*), indicating how they were calculated

Our web collection on statistics for biologists contains articles on many of the points above.

Software and code

Policy information about availability of computer code

Data collection	Orbitrap Fusion Tribrid mass spectrometer (Thermo Xcalibur Xcalibur 4.1); BD LSR Fortessa (BD FACSDiva 8.0); Illumina Nextseq 500 sequencer (NextSeq Control Software v2)
Data analysis	NGS data: STAR (version 2.5.4), EdgeR (version 3.26.8), Homer (version 4.10.3)<br>Mass spectrometry data: MaxQuant (version 1.6.17.0), Perseus (version 1.6.14.0)

For manuscripts utilizing custom algorithms or software that are central to the research but not yet described in published literature, software must be made available to editors and reviewers. We strongly encourage code deposition in a community repository (e.g. GitHub). See the Nature Portfolio guidelines for submitting code & software for further information.

Data

Policy information about availability of data

All manuscripts must include a data availability statement. This statement should provide the following information, where applicable:

- Accession codes, unique identifiers, or web links for publicly available datasets
- A description of any restrictions on data availability
- For clinical datasets or third party data, please ensure that the statement adheres to our policy

All primary data are available from the corresponding author. The sequencing data have been deposited at the GEO database (accession GSE217524, https://

www.ncbi.nlm.nih.gov/geo/query/acc.cgi?acc=GSE217524). The mass spectrometry data have been deposited at the PRIDE database (accession PXD037854, http://www.ebi.ac.uk/pride/archive/projects/PXD037854).

Human research participants

Policy information about studies involving human research participants and Sex and Gender in Research.

Reporting on sex and gender	Not applicable
Population characteristics	Not applicable
Recruitment	Not applicable
Ethics oversight	Not applicable

Note that full information on the approval of the study protocol must also be provided in the manuscript.

Field-specific reporting

Please select the one below that is the best fit for your research. If you are not sure, read the appropriate sections before making your selection.

☒ Life sciences ☐ Behavioural & social sciences ☐ Ecological, evolutionary & environmental sciences

For a reference copy of the document with all sections, see nature.com/documents/nr-reporting-summary-flat.pdf

Life sciences study design

All studies must disclose on these points even when the disclosure is negative.

Sample size	For cell culture experiments, sample sizes were variable depending on sample availability. For animal experiments, the group size was determined based on the statistical analysis performed by a statistician scientist, Dr. Serna Higuita (Institute of Clinical Epidemiology and Applied Biometrics, University Hospital Tübingen), in a predefined procedure at the University of Tübingen. The group size is determined based on the anticipated effect size and average effect sizes from previous experiments. In our study, the specified group size was sufficient to report siginificant differences.
Data exclusions	No data were excluded from the analysis.
Replication	Most of the experiments were replicated; the number of replicates is indicated in figure legends.
Randomization	For animal experiments, mice of the same sex were randomly assigned to experimental groups. For cell culture experiments, we compared cells of two genetic backgrounds or cells of the same background with and without treatment (e.g., hydroxyurea). Within each group, analyzed cells are selected from a large population in a random manner intrinsically (e.g., immunoblotting or immunoprecipitation experiments) or by random allocation of the imaged area (e.g., immunofluorescence and PLA assays), followed by software-based quantification of signal intensities and foci.
Blinding	The investigators were not blinded to group allocation during data collection or analysis, due to the unbiased nature of molecular biological assays used (please see above under Randomization) and computer-based quantification of the effects.

Reporting for specific materials, systems and methods

We require information from authors about some types of materials, experimental systems and methods used in many studies. Here, indicate whether each material, system or method listed is relevant to your study. If you are not sure if a list item applies to your research, read the appropriate section before selecting a response.

Materials & experimental systems

n/a	Involved in the study	
☐	☒	Antibodies
☐	☒	Eukaryotic cell lines
☒	☐	Palaeontology and archaeology
☐	☒	Animals and other organisms
☒	☐	Clinical data
☒	☐	Dual use research of concern

Methods

n/a	Involved in the study	
☐	☒	ChIP-seq
☐	☒	Flow cytometry
☒	☐	MRI-based neuroimaging

Antibodies

Antibodies used

Immunoblotting (dilution 1:1000 for all primary antibodies and 1:5000 for secondary antibodies)
Histone H4 (L64C1), Cell Signaling 2935.
Vinculin (V824), Sigma SAB4200080.
beta-Actin (AC-15), Santa Cruz sc-69879.
Cdc6 (C42F7), Cell Signaling 3387.
Mcm3 (E-8), Santa Cruz sc-390480.
Mcm5. Abcam ab17967.
Mcm6 (H-8), Santa Cruz sc-393618.
Mcm7, Bethyl A302-585A.
c-Myc (D3N8F), Cell Signaling 13987.
Emerin (H-12), Santa Cruz sc-25284.
pS824-Kap1, Bethyl A300-767A.
pS317-Chk1, Cell Signaling 12302.
pS4/8-Rpa2, Bethyl a300-245A.
RNAPII (D8L4Y), Cell Signaling 14958.
pS2-RNAPII Abcam ab5095.
pS5-RNAPII (D9N5I), Cell Signaling 13523.
Rrm2 (A-5) Santa Cruz sc-398294.
Ercc1 (D-10), Santa Cruz sc-17809.
Ini1 (A-5), Santa Cruz sc-166165.
GST Tag, Bethyl A190-122A.
anti-rabbit IgG HRP conjugate. Cell Signaling 7074.
anti-mouse IgG, HRP conjugate. Cell Signaling 7076.

Immunofluorescence and PLA (dilution 1:100 for all antibodies)
E2f4, Proteintech 10923-1-AP.
Biotin, Santa Cruz sc-101339.
Trim33, Sigma HPA004345.
Recql, Bethyl A300-447.
Recql (A-9), Santa Cruz sc-166388.
Mcm2 (D7G11), Cell Signaling 3619.
PCNA (PC-10), Santa Cruz sc-56.
53bp1, Novus NB100-304.
pS2-RNAPII, Abcam ab5095.
RNAPII (D8L4Y), Cell Signaling 14958.
pS139-H2AX (20E3), Cell Signaling 9718.
pH2AX (ser139), Santa Cruz sc-517348.

Immmunihistochemistry
Trim33, Sigma HPA004345; dilution 1:50.
pS139-H2AX (20E3), Cell Signaling 9718; dilution 1:100.
CD3 (SP7), Thermo Scientific RM-9107; dilution 1:100.

Immunoprecipitation, 1-2ug antibody / sample
E2f4 (GG22-2A6), Sigma 05-312.
E2f4, Proteintech 10923-1-AP.
FLAG (M2), Sigma F3165.
Recql, Abcam ab151501.
Recql, Invitrogen PA5-27100.
Trim33 (6D1). Sigma WH0051592M1.

ChIP and CUT&RUN, 2-5 ug antibody / sample
E2f4, Proteintech 10923-1-AP.
HA tag (6E2), Cell Signaling 2367.
Recql, Abcam ab151501.

Validation

Histone H4 (L64C1), Cell Signaling 2935. Supplier validation: "REACTIVITY - H M R Mk; Application - Western Blotting".
Vinculin (V824), Sigma SAB4200080. Supplier validation: "species reactivity - rabbit, chicken, human, rat, monkey, mouse, canine; technique(s) - immunocytochemistry, immunoprecipitation (IP): suitable, western blot: 0.005-0.01 µg/mL".
b-Actin (AC-15), Santa Cruz sc-69879. Supplier validation: "is recommended for detection of b-Actin of broad species origin by Western Blotting".
Cdc6 (C42F7) Cell Signaling 3387. Supplier validation: "REACTIVITY - H M R Hm Mk; Application - Western Blotting".
Mcm3 (E-8), Santa Cruz sc-390480. Supplier validation: "is recommended for detection of MCM3 of mouse, rat and human origin by Western Blotting".
Mcm5. Abcam ab17967. Supplier validation: "Suitable for: WB, IHC-P; Reacts with: Human".
Mcm6 (H-8), Santa Cruz sc-393618. Supplier validation: "is recommended for detection of MCM6 of mouse, rat and human origin by Western Blotting".
GST Tag, Bethyl A190-122A. Supplier validation: "Applications - Western Blot (WB); Species reactivity - Tag".
CD3 (SP7), Thermo Scientific RM-9107. Supplier validation: "Application - Immunohistochemistry". Species validation by the CMCP facility, Pathological Institute, University Hospital Heidelberg.
Mcm7, Bethyl A302-585A. Supplier validation: "Applications - Western Blot; Species Reactivity - Human".
c-Myc (D3N8F), Cell Signaling 13987. Supplier validation: "REACTIVITY H M R Mk; Application - Western Blotting".

Emerin (H-12), Santa Cruz sc-25284. Supplier validation: " is recommended for detection of emerin of human origin by Western Blotting".
pH2AX (ser139), Santa Cruz sc-517348. Supplier validation: "Recommended for detection of Ser 139 phosphorylated Histone H2A.X of mouse, rat and human origin by Western Blotting, immunoprecipitation, immunofluorescence)".
pS139-H2AX (20E3), Cell Signaling 9718. Supplier validation: "REACTIVITY - H M R Mk; Application - Western Blotting, Immunohistochemistry, Immunofluorescence)".
pS824-Kap1, Bethyl A300-767A. Supplier validation: "Species Reactivity - Human, Mouse; Applications - Western Blot (WB)".
pS317-Chk1, Cell Signaling 12302. Supplier validation: "REACTIVITY - H M Mk; Application - Western Blotting".
pS4/8-Rpa2, Bethyl a300-245A. Supplier validation: "Application - WB, IP, IHC, ICC-IF; Species reactivity - human, mouse".
RNAPII (D8L4Y), Cell Signaling 14958. Supplier validation: "REACTIVITY H M R Mk; Application - Western Blotting, Chromatin IP, Chromatin IP-seq".
pS2-RNAPII, Abcam ab5095. Supplier validation: "Suitable for: ELISA, WB, IHC-P, ICC/IF; Reacts with: Mouse, Rat, Human, Saccharomyces cerevisiae".
pS5-RNAPII (D9N5I), Cell Signaling 13523. Supplier validation: "REACTIVITY H M R Mk; Application - Western Blotting, Immunoprecipitation, Chromatin IP, Chromatin IP-seq, CUT&RUN".
Rrm2 (A-5) Santa Cruz sc-398294. Supplier validation: "is recommended for detection of R2 of mouse, rat und human origin by WB, IP, IF, IHC(P) and ELISA".
Ercc1 (D-10), Santa Cruz sc-17809. Supplier validation: "is recommended for detection of ERCC1 of mouse, rat and human origin by Western Blotting, immunofluorescence".
Ini1 (A-5), Santa Cruz sc-166165 is recommended for detection of Ini1 of mouse, rat and human origin by Western Blotting, immunoprecipitation, immunofluorescence".
E2f4, Proteintech 10923-1-AP. Supplier validation: "Reactivity - human, mouse, rat. Applications - WB, IP, IHC, ChIP, ELISA".
Trim33 Sigma HPA004345. Supplier validation: "technique(s) - immunoblotting, immunofluorescence, immunohistochemistry". In lab species validation using knockout cells in IF, IHC and IB".
Recql, Bethyl A300-447. Supplier validation: "Species Reactivity - Human, Mouse; Applications: Western Blot (WB), Immunoprecipitation (IP), Immunohistochemistry (IHC)".
Recql (A-9), Santa Cruz sc-166388. Supplier validation: "RecQL1 (A-9) is recommended for detection of RecQL1 of mouse, rat and human origin by Western Blotting, immunoprecipitation, immunofluorescence".
Mcm2 (D7G11), Cell Signaling 3619. REACTIVITY H M R Mk; Application - Western Blotting , Immunofluorescence (Immunocytochemistry)".
PCNA (PC-10), Santa Cruz sc-56. Supplier validation: " is recommended for detection of PCNA of mouse, rat, human, insect and S. pombe origin by Western Blotting, immunoprecipitation, immunofluorescence".
53bp1, Novus NB100-304. Supplier validation: "Reactivity - Hu, Mu, etc.; Applications - WB, ChIP, Flow, Flow-IC, IB, ICC/IF".
E2f4 (GG22-2A6), Sigma 05-312. Supplier validation: "species reactivity - rat, mouse, human; technique(s) - ChIP: suitable (ChIP-seq) immunoprecipitation (IP): suitable, western blot: suitable".
FLAG (M2), Sigma F3165. Supplier validation: "species reactivity - all; technique(s) western blot".
Recql, Abcam ab151501. Supplier validation: "Suitable for: WB, IHC-P, IP; Reacts with: Mouse, Human".
Recql, Invitrogen PA5-27100. Supplier validation: "Applications - Western Blot (WB), Immunoprecipitation (IP)Immunocytochemistry (ICC/IF); Species Reactivity - Human, Mouse".
Trim33 (6D1). Sigma WH0051592M1. Supplier validation: "technique(s) - indirect immunofluorescence: suitable, western blot: 1-5 µg/mL; species reactivity - human". Validated in the lab for IP and WB using murine knockout cells.
HA tag (6E2). Cell Signaling 2367. Supplier validation: "REACTIVITY - All; Application Western Blotting, Immunohistochemistry, Immunofluorescence, Flow Cytometry (Fixed/Permeabilized)".

Eukaryotic cell lines

Policy information about cell lines and Sex and Gender in Research

Cell line source(s)	p19KO/Nras, p19KO/Nras/Myc- provided by Dr. Daniel Dauch (University Hospital Tübingen); not available commercially. LentiX (HEK293T) - provided by Michael Hudecek (University Hospital Würzburg); available from Takara Bio. HeLa, U2OS - provided by Martin Eilers (University of Würzburg); available from ATCC.
Authentication	HeLa and U2OS cells were authenticated by STR typing. The other cell lines were not authenticated.
Mycoplasma contamination	All cell lines tested negative for mycoplasma contamination
Commonly misidentified lines (See ICLAC register)	No commonly misidentified cell lines were used

Animals and other research organisms

Policy information about studies involving animals; ARRIVE guidelines recommended for reporting animal research, and Sex and Gender in Research

Laboratory animals	Mus musculus, C57Bl6/J, 4-6 weeks of age.
Wild animals	No wild animals were used in the study.
Reporting on sex	Equal number of male and female animals were used per group.
Field-collected samples	No field-collected samples were used in the study.

Ethics oversight | Regional government of Tübingen (approval nr. M09/20G)

Note that full information on the approval of the study protocol must also be provided in the manuscript.

ChIP-seq

Data deposition

☒ Confirm that both raw and final processed data have been deposited in a public database such as GEO.

☒ Confirm that you have deposited or provided access to graph files (e.g. BED files) for the called peaks.

Data access links<br>*May remain private before publication.*	GEO portal, GSE217524 (https://www.ncbi.nlm.nih.gov/geo/query/acc.cgi?acc=GSE217524)
Files in database submission	Trim33KO_E2f4.bedGraph.gz, Trim33KO_E2f4.fastq.gz, Trim33KO_input.bedGraph.gz, Trim33KO_input.fastq.gz, Trim33WT_E2f4.bedGraph.gz, Trim33WT_E2f4.fastq.gz, Trim33WT_input.bedGraph.gz, Trim33WT_input.fastq.gz
Genome browser session<br>(e.g. UCSC)	The bedGraph files are provided for vizualization using a genome browser

Methodology

Replicates	Single replicate per sample
Sequencing depth	at least 60 million reads per sample
Antibodies	E2f4 (Proteintech, 10923-1-AP)
Peak calling parameters	Reads were mapped using STAR; begraph files were generated using Homer; peaks were called using Homer's FindPeaks command with default parameters
Data quality	FastQC was used to assess quality of ChIP-seq reads. Peak calling using Homer FindPeaks yielded 237 peaks in Trim33-WT cells and 2269 peaks in Trim33-KO cells
Software	STAR (version 2.5.4)<br>Homer (version 4.10.3)

Flow Cytometry

Plots

Confirm that:

☒ The axis labels state the marker and fluorochrome used (e.g. CD4-FITC).

☐ The axis scales are clearly visible. Include numbers along axes only for bottom left plot of group (a 'group' is an analysis of identical markers).

☒ All plots are contour plots with outliers or pseudocolor plots.

☒ A numerical value for number of cells or percentage (with statistics) is provided.

Methodology

Sample preparation	Cells were collected by trypsinzation, fixed with ethanol and stained with propidium iodide
Instrument	BD LSR Fortessa
Software	BD FACSDiva v8.0, Flowing v2.5.1
Cell population abundance	No cell sorting was performed
Gating strategy	Samples were gated based on FSC/SSC to exclude debris before measuring the distribution of PI-positive cells

☐ Tick this box to confirm that a figure exemplifying the gating strategy is provided in the Supplementary Information.
